# Activities of daily living predict periprocedural myocardial infarction and injury following percutaneous coronary intervention: a cross-sectional study

**DOI:** 10.1186/s12872-023-03468-5

**Published:** 2023-08-29

**Authors:** Yifan Pan, Min Xu, Yaosheng Mei, Yunxiang Wang, Qiongli Zhang

**Affiliations:** 1 Department of Cardiology, Yongkang People’s Hospital, Yongkang, Zhejiang 321300 China; 2 Department of Cardiology, Yongkang Hospital, Yongkang, Zhejiang 321300 China

**Keywords:** Activities of daily living, Barthel index, Periprocedural myocardial infarction, Periprocedural myocardial injury

## Abstract

**Background:**

In recent years, there has been growing interest in exploring the relationship between activities of daily living (ADL) and cardiovascular diseases. This retrospective cross-sectional study aimed to investigate the association of ADL measured by Barthel index (BI) with periprocedural myocardial infarction (PMI) and injury following percutaneous coronary intervention (PCI).

**Methods:**

Enrolled patients were stratified into impaired and unimpaired ADL groups according to their BI scores. Logistic regressions were conducted to explore the association of ADL on admission with periprocedural myocardial injury and infarction. Restricted cubic spline (RCS) curve and subgroup analysis were also performed.

**Results:**

Totally, 16.4% of patients suffered from PMI; the mean age was 65.8 ± 10.4 years old. RCS analysis showed that the morbidity of periprocedural myocardial infarction and injury showed a downward tendency with increasing BI scores. Multivariable logistic regression analysis demonstrated that impaired ADL was an independent risk factor for periprocedural myocardial infarction (OR = 1.190, 95% CI [1.041, 1.360], *P* = 0.011) and injury (OR = 1.131, 95% CI [1.017, 1.257], *P* = 0.023). Subgroup analysis showed that the association between ADL and PMI was founded in several subgroups, while the association between ADL and periprocedural myocardial injury was founded only in BMI ≥ 24 kg/m^2^ subgroup.

**Conclusion:**

Impaired ADL at hospital admission was an independent risk factor for periprocedural myocardial infarction and injury among patients following PCI.

**Supplementary Information:**

The online version contains supplementary material available at 10.1186/s12872-023-03468-5.

## Introduction

Coronary artery disease (CAD) is a leading cause of mortality worldwide [1, 2]. Currently, percutaneous coronary intervention (PCI) is a widely recognized and well-established therapeutic approach for treating CAD and could significantly reduce CAD mortality [3]. Despite the significant progress in PCI procedures and techniques in recent decades, there still exists a high incidence of periprocedural myocardial infarction (known as type 4a myocardial infarction) and injury, affecting up to 40% of patients [4, 5]. Periprocedural myocardial injury and infarction are related to myocardial damage following PCI, leading to worsened cardiac outcomes [6, 7]. Therefore, establishing a simple and cost-effective predictive model to identify high-risk groups susceptible to periprocedural myocardial infarction and injury is of utmost importance. Previous studies have attempted to identify predictive factors associated with periprocedural myocardial infarction and injury, resulting in some significant results. These risk factors encompass advanced age, renal failure, chronic total occlusion (CTO), left main disease, and multi-vessel PCI [3, 8–10]. Several studies have recognized the neutrophil/lymphocyte ratio, stress level, syntax score (SS), and syntax score II (SSII) as potential predictors of PCI outcomes [11–14]. However, limited attention has been given to the influence of a patient’s physical ability on the incidence of periprocedural myocardial infarction and injury [15, 16].

As the global population continues to age, there has been a growing focus on individual capacity to perform everyday tasks, known as activities of daily living (ADL), in clinical settings. The Barthel index (BI), a highly reliable and valid tool, is commonly employed to assess a patient’s ability to perform necessary ADLs during the rehabilitation process [17, 18]. A 2-year follow-up study revealed that ADL assessment at hospital admission could be used for risk assessment in patients with acute coronary syndrome (ACS) and long-term treatment planning [19]. Another recent study demonstrated that ADL assessment at discharge was an independent risk factor for mortality in patients with acute myocardial infarction (AMI) [20]. However, the association between ADL and short-term complications of cardiovascular disease has not been thoroughly investigated. Furthermore, the association between ADL and periprocedural myocardial infarction and injury remains unclear.

Thus, this retrospective study was conducted to assess whether ADL at admission could predict periprocedural myocardial infarction and injury, and to offer suitable preventive and management strategies for patients undergoing PCI.

## Methods

### Study population

**Fig. 1:**
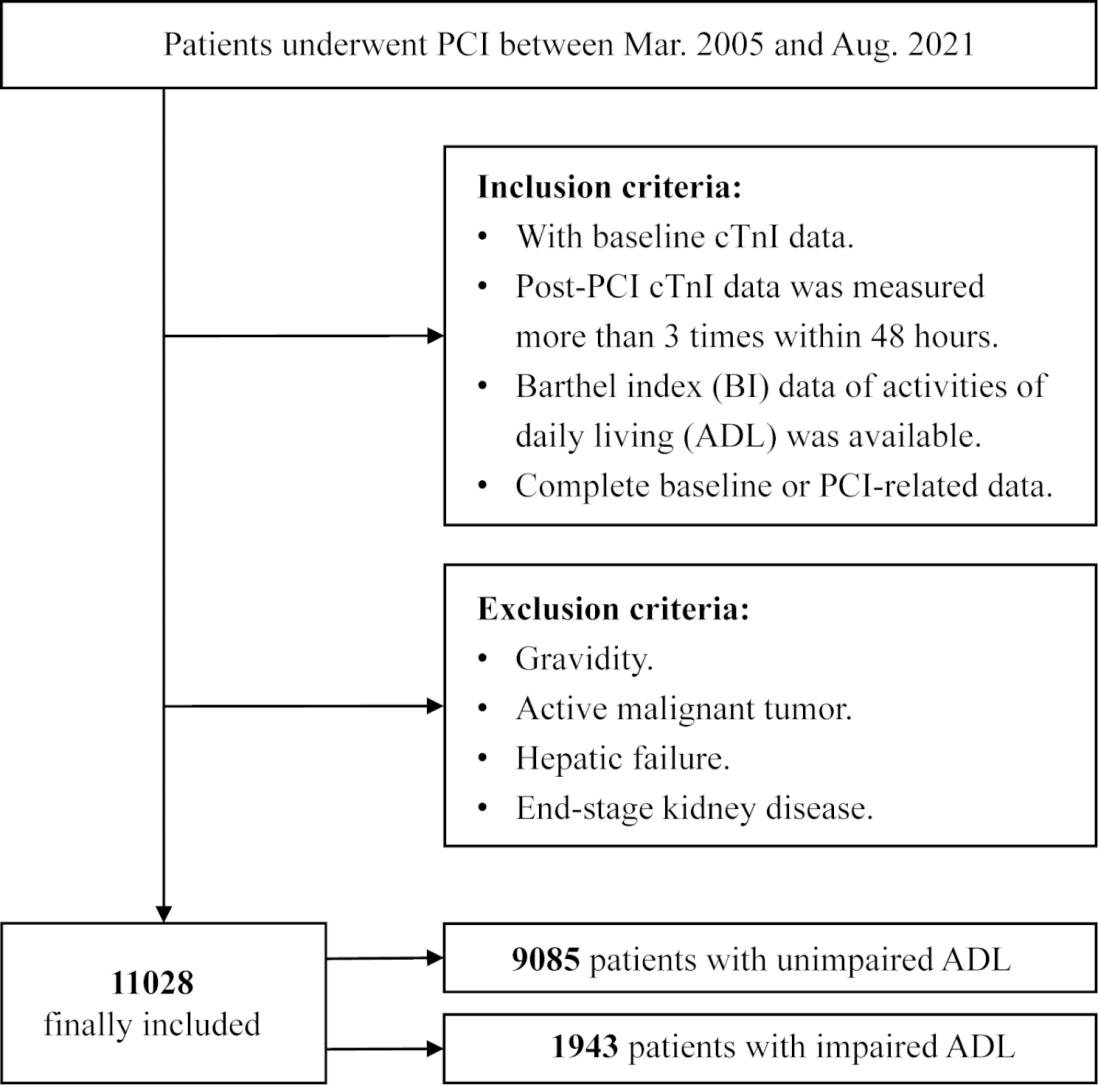
Flow chart for study design. PCI, percutaneous coronary intervention; cTnI, Cardiac troponin I; ADL, Activities of daily living; BI, Barthel Index

This study was a retrospective review of all consecutive eligible patients following PCI from March 2005 to August 2021 at Sir Run Run Shaw Hospital and its medical consortium hospitals. Additional details about the study subjects are listed in Fig. 1. The inclusion criteria were: (1) patients with BI scores assessed at hospital admission; (2) patients with documented cardiac troponin I (cTnI) before PCI and at 8, 16, 24, and 48 h after PCI; (3) patients with complete data of demographics, angiographic procedure, laboratory examination, and medication. Exclusion criteria were (1) pregnancy, (2) active malignant tumor, (3) hepatic failure, (4) heart failure, and (5) end-stage kidney disease. Finally, 11,028 patients were enrolled in the study, which adhered to the principles outlined in the Declaration of Helsinki and was approved by the Ethics Committee of Sir Run Run Shaw Hospital.

### Data collection and assessment of clinical parameters

Various parameters involving patient demographics, clinical features, laboratory results, medications, and angiographic characteristics were extracted from the hospital information system.

**Fig. 2:**
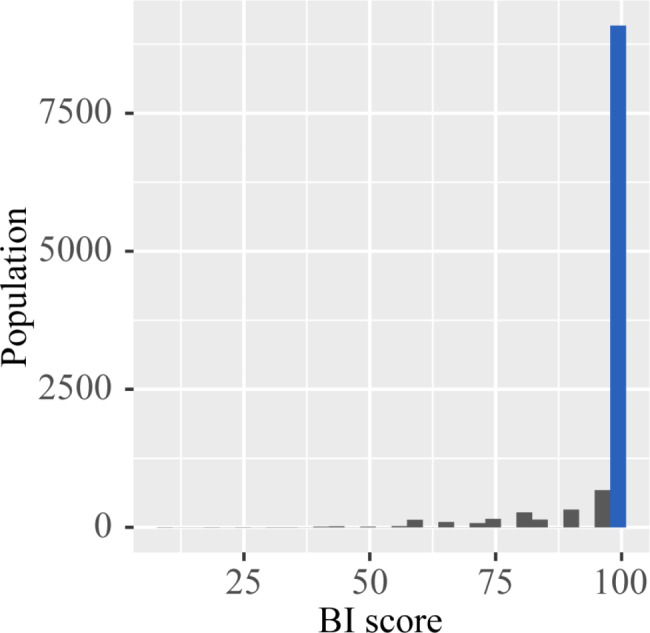
The population distribution of BI scores. The grey histograms described the overall distribution in each BI group. Horizontal axis, BI (scores); left axis, population count (persons); BI, Barthel index

Healthcare professionals routinely evaluate BI score upon hospital admission. The BI score was recommended as an assessment of ADL. The BI score consists of 10 items about feeding (0, 5), grooming (0, 5), bathing (0, 5), dressing (0, 5, 10), bladder control (0, 5, 10), toilet use (0, 5, 10), bowel control (0, 5, 10), ambulating (0, 5, 10, 15), chair transferring (0, 5, 10, 15), and stair climbing (0, 5, 10, 15) [21]. The overall score ranged from 0 (completely dependent) to 100 (completely independent). Figure 2 displayed the population distribution of BI scores. The whole population was divided into two groups: the unimpaired ADL group (BI score = 100) and the impaired ADL group (BI score < 100).

Based on the latest revised fourth universal definition of myocardial infarction (UDMI) published in 2018, periprocedural myocardial infarction was defined in this study as an increase in cTnI levels greater than 5 times of the upper reference limit (URL) within 48 h following PCI, in addition to one or more of the following: (1) the clinical criteria, manifested with prolonged chest pain attributed to ischemic changes (≥ 20 min); (2) ECG criteria, including ischemic ST changes or new pathological Q waves; or (3) imaging criteria, manifested with imaging evidence of new loss of viable myocardium or new regional wall motion abnormality or angiographic evidence of a flow-limiting complication [22]. Periprocedural myocardial injury was defined as an increase in cTnI levels 1 time greater than the URL [23]. cTnI fold-elevation referred to the post-PCI cTnI fold elevated from the URL.

### Statistical analysis

Statistical analyses were performed using SPSS software version 22.0 (SPSS Inc., Chicago, IL, United States) and R version 4.0.5 (The R Foundation for Statistical Computing, Vienna, Austria). Continuous variables were expressed as mean ± standard deviation (SD) and compared using T-test in case of normal distribution, while expressed as median (interquartile range [IQR]) and compared using Mann-Whitney U-test in case of non-normal distribution. Categorical variables were expressed as counts (percentage) and compared using Chi-Square test or Fisher’s exact test.

Univariant and multivariate logistic regression were used to determine the association of the ADL at hospital admission with periprocedural myocardial infarction and injury. The previously recognized risk factors variables of periprocedural myocardial infarction and injury from previous studies were included as covariates in the multivariate regression analysis [6], detailed descriptions of specific measures administered were given below: Model 1 adjusted for none; Model 2 adjusted for age (years old), renal dysfunction (eGFR ≥ 60 or < 60 ml/min/1.73 m^2^), NT-proBNP (×10^3^pg/mL); Model 3 based on Model 2, additionally adjusted for CTO (yes or no), calcification (yes or no), rotational atherectomy (yes or no), LMPCI (yes or no), multivessel PCI (yes or no) and total stent length (mm). Restricted cubic spline (RCS) curve according to Model 3 correction was performed for visualization analysis. Based on age (≥ 65 or < 65 years old), ever smoked (yes or no), BMI (≥ 24 or < 24 kg/m^2^) and eGFR (≥ 60 or < 60 ml/min/1.73m^2^), subgroup analysis was performed to test whether the association of the ADL with the occurrence of periprocedural myocardial injury and infarction was constant.

## Results

### Baseline characteristics

11,028 patients were enrolled, and the mean age was 65.8 ± 10.4 years old. Patients with impaired ADL accounted for 17.6%. The incidence of periprocedural myocardial injury was 46.6% (5,137/11,028), and periprocedural myocardial infarction was 16.4% (1,808/11,028).

Table 1 showed the details of the baseline clinical and procedural characteristics. Periprocedural myocardial infarction patients had significantly lower scores of BI (96.1 ± 10.1 vs. 97.1 ± 8.4, P < 0.001) and higher proportions of impaired ADL (22.2% vs. 17.1%, *P* < 0.001); higher cTnI fold-elevation, higher levels of baseline white blood cell (WBC), C-reactive protein (CRP), N-terminal pro-brain natriuretic peptide (NT-proBNP), and lower levels of estimated glomerular filtration rate (eGFR) and platelet counts (all *P* values < 0.05) also were noted among this group of patients in comparison with patients without occurring periprocedural myocardial infarction. In terms of vessel conditions and procedure data, periprocedural myocardial infarction patients had higher proportions of calcification (23.9% vs. 14.6%, *P* < 0.001), CTO (16.1% vs. 9.4%, *P* < 0.001), rotational atherectomy (5.1% vs. 1.0%, *P* < 0.001), LM PCI (7.7% vs. 4.3%, *P* < 0.001), multivessel PCI (14.5% vs. 8.5%, *P* < 0.001), and longer total stent length (48.0 [28.0, 70.0] vs. 29.0 [18.0, 48.0] mm, *P* < 0.001). The medication characteristics of periprocedural myocardial infarction patients were reflected in a higher proportion of use of angiotensin converting enzyme inhibitor/angiotensin receptor antagonist (ACEI/ARB), beta-blockers, calcium channel blockers (CCB) (all *P* values < 0.05).

**Table 1:** Baseline characteristics

Characteristics	Overall (n = 11,028)	Periprocedural Myocardial Infarction		*P* value
		No (n = 9220)	Yes (n = 1808)	
**Demographic features**				
Age, years old	65.82 ± 10.41	65.74 ± 10.41	66.21 ± 10.37	0.075
BMI, kg/m^2^	24.58 ± 3.28	24.54 ± 3.26	24.77 ± 3.36	0.006*
Male, n (%)	7854 (71.2)	6580 (71.4)	1274 (70.5)	0.456
Ever smoked, n (%)	4098 (37.2)	3438 (37.3)	660 (36.5)	0.546
Diabetes, n (%)	3213 (29.1)	2696 (29.2)	517 (28.6)	0.600
Hypertension, n (%)	7496 (68.0)	6245 (67.7)	1251 (69.2)	0.235
Barthel index, scores	96.98 ± 8.71	97.14 ± 8.39	96.14 ± 10.14	< 0.001*
impaired ADL, n (%)	1943 (17.6)	1552 (16.8)	391 (21.6)	< 0.001*
**PCI procedure data**				
LM PCI, n (%)	535 (4.9)	395 (4.3)	140 (7.7)	< 0.001*
LAD PCI, n (%)	5449 (49.4)	4489 (48.7)	960 (53.1)	0.001*
LCX PCI, n (%)	2188 (19.8)	1751 (19.0)	437 (24.2)	< 0.001*
RCA PCI, n (%)	2952 (26.8)	2557 (27.7)	395 (21.8)	< 0.001*
Multivessel PCI, n (%)	1043 (9.5)	780 (8.5)	263 (14.5)	< 0.001*
Rotational atherectomy, n (%)	180 (1.6)	88 (1.0)	92 (5.1)	< 0.001*
CTO, n (%)	1162 (10.5)	871 (9.4)	291 (16.1)	< 0.001*
Calcification, n (%)	1775 (16.1)	1343 (14.6)	432 (23.9)	< 0.001*
Total stent length, mm	30.0 [18.0, 53.0]	29.0 [18.0, 48.0]	48.0 [28.0, 70.0]	< 0.001*
**Laboratory data**				
cTnI fold-elevation	0.9 [0.1, 2.9]	0.6 [0.0, 1.6]	10.4 [6.8, 21.6]	< 0.001*
WBC, ×10^9^/L	6.4 ± 1.8	6.4 ± 1.8	6.7 ± 2.0	< 0.001*
LDL-C, mmol/L	1.97 [1.50, 2.62]	1.98 [1.50, 2.62]	1.96 [1.50, 2.64]	0.835
CRP, mg/L	1.3 [0.6, 3.2]	1.3 [0.6, 3.0]	1.6 [0.7, 4.0]	< 0.001*
HbA1c, %	6.1 [5.6, 6.8]	6.1 [5.6, 6.8]	6.1 [5.6, 6.9]	0.075
eGFR, mL/(min×1.73m^2^)	87.0 [72.1, 97.7]	87.9 [73.0, 98.0]	83.4 [66.2, 95.2]	< 0.001*
Platelet, ×10^9^/L	190.9 ± 57.6	191.3 ± 57.4	188.7 ± 58.7	0.081
NT-proBNP, pg/mL	131.0 [57.0, 408.0]	121.0 [54.0, 365.0]	207.1 [77.0, 691.3]	< 0.001*
**Medication**				
ACEI/ARB, n (%)	5583 (50.6)	4625 (50.2)	958 (53.0)	0.030*
BB, n (%)	5493 (49.8)	4537 (49.2)	956 (52.9)	0.005*
CCB, n (%)	4120 (37.4)	3353 (36.4)	767 (42.4)	< 0.001*
Statin, n (%)	10,849 (98.4)	9064 (98.3)	1785 (98.7)	0.234

Categorical data are presented as n (%) and continuous data are expressed as mean ± standard deviation or median [interquartile range]. BMI, Body mass index; ADL, Activities of daily living; PCI, Percutaneous coronary intervention; LM, Left main; LAD, Left anterior descending branch; LCX, Left circumflex artery; RCA, Right coronary artery; CTO, Chronic total occlusion; cTnI, Cardiac troponin I; WBC, White blood cell; LDL-C, Low-density lipoprotein cholesterol; CRP, C-reactive protein; HbAlc, Hemoglobin A1c; eGFR, estimated glomerular filtration rate; NT-proBNP, N-terminal pro-brain natriuretic peptide ACEI, Angiotensin converting enzyme inhibitor; ARB, Angiotensin receptor antagonist; BB, Beta-blockers; CCB, Calcium channel blocker.*P < 0.05

The significance of the intergroup differences in the above variables could be observed almost immediately when grouping by whether periprocedural myocardial injury had occurred or not (Table S1). Patients with periprocedural myocardial injury incidence had a significantly higher proportion of impaired ADL than those without (19.7% vs. 15.8%, *P* < 0.001).

### The association of the ADL with the incidence of periprocedural myocardial injury and infarction

**Fig. 3:**
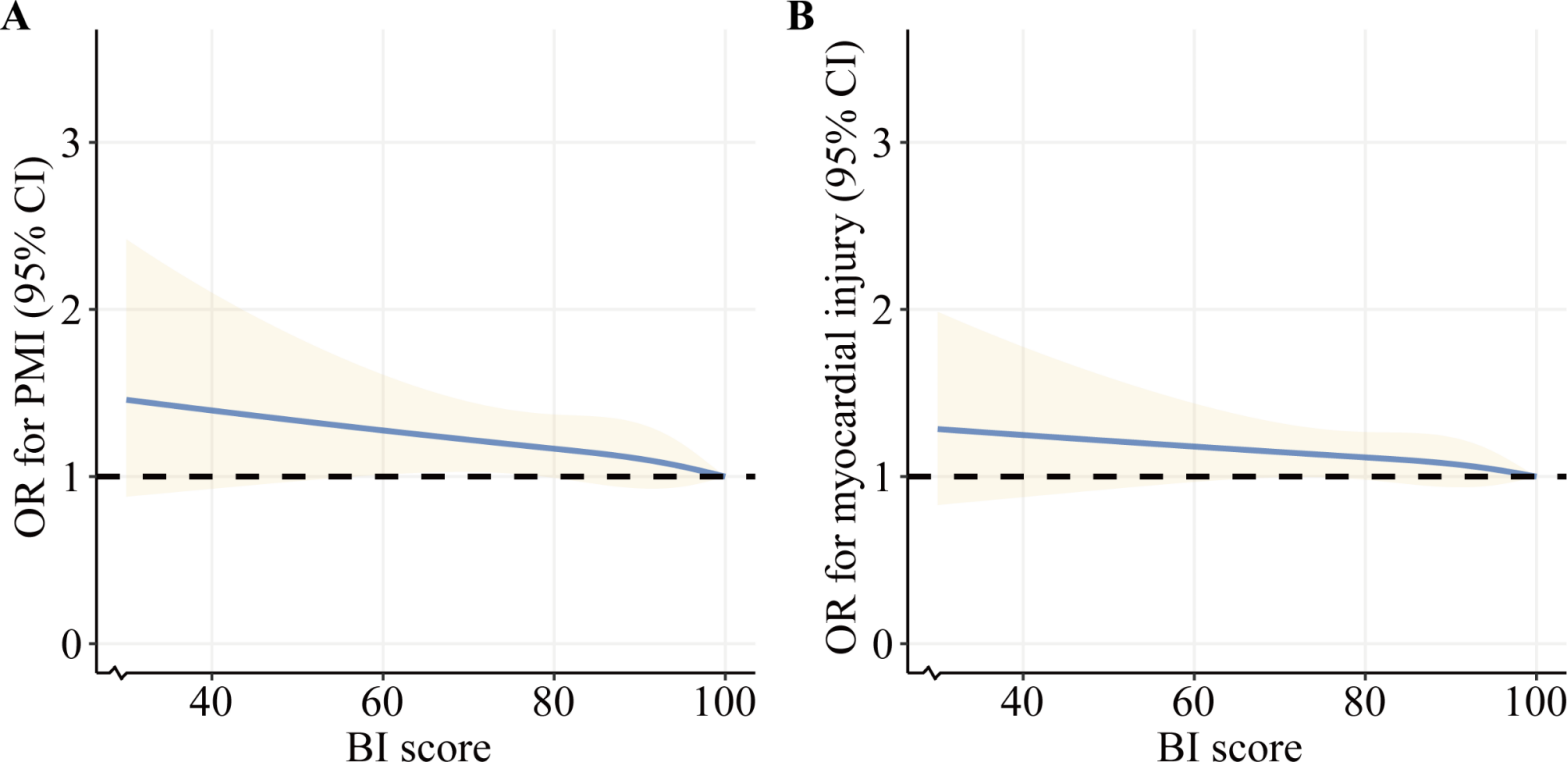
**(A)** Restricted cubic spline analysis for exploring the relationship between BI scores and periprocedural myocardial infarction. **(B)** Restricted cubic spline analysis for exploring the relationship between BI scores and periprocedural myocardial injury. The blue solid line showed the adjusted odds ratio, and the yellow shadow area around the solid line indicated 95% confidence interval. BI, Barthel index

As shown in Fig. 3, RCS analysis was conducted to explore the relationship of BI scores with periprocedural myocardial injury and infarction. Along with BI scores increasing, periprocedural myocardial injury and infarction incidence showed a downward tendency.

The study used univariable and multivariable logistic regression analysis to investigate the association between ADL and periprocedural myocardial infarction incidence (Table 2). Model 1 shows that impaired ADL was a risk factor for periprocedural myocardial infarction (OR = 1.363, 95% CI [1.204, 1.544], *P* < 0.001). After adjusting for age, renal dysfunction, and NT-proBNP (shown in Model 2), and further adjusting for vessel conditions and procedure data (CTO, calcification, rotational atherectomy, LM PCI, multivessel PCI, and total stent length) (shown in Model 3), multivariable linear regression analysis demonstrated that impaired ADL was an independent risk factor for the incidence of periprocedural myocardial infarction (Model 2: OR = 1.210, 95% CI [1.064, 1.377], *P* = 0.004; Model 3: OR = 1.190, 95% CI [1.041, 1.360], *P* = 0.011).

**Table 2:** Logistic regression analyses of ADL on periprocedural myocardial infarction

	Variables	OR [95% CI]	*P* value
**Model 1**			
	impaired ADL	1.363 [1.204, 1.544]	< 0.001*
**Model 2**			
	impaired ADL	1.210 [1.064, 1.377]	0.004*
	Age	1.099 [0.992, 1.219]	0.072
	Renal dysfunction	1.331 [1.150, 1.540]	< 0.001*
	NT-proBNP	1.118 [1.080, 1.157]	< 0.001*
**Model 3**			
	impaired ADL	1.190 [1.041, 1.360]	0.011*
	Age	1.122 [1.009, 1.248]	0.034*
	Renal dysfunction	1.263 [1.086, 1.470]	0.002*
	NT-proBNP	1.096 [1.058, 1.136]	< 0.001*
	CTO	1.349 [1.155, 1.574]	< 0.001*
	Calcification	1.295 [1.129, 1.485]	< 0.001*
	Rotational atherectomy	3.261 [2.351, 4.522]	< 0.001*
	LM PCI	1.384 [1.111, 1.723]	0.004*
	Multivessel PCI	1.318 [1.117, 1.555]	0.001*
	Total stent length	1.017 [1.015, 1.019]	< 0.001*

Model 1 adjusted for none

Model 2 adjusted for age (years old), renal dysfunction (eGFR ≥ 60 or < 60 ml/min/1.73 m^2^), NT-proBNP (×10^3^pg/mL)

Model 3 based on Model 2, additionally adjusted for CTO (yes or no), calcification (yes or no), rotational atherectomy (yes or no), LM PCI (yes or no), multivessel PCI (yes or no) and total stent length (mm)

OR, Odds ratio; CI, Confidence interval. Other abbreviations as in Table 1.*P < 0.05

Besides, multivariable logistic regression analysis also confirmed that age (OR = 1.122, 95% CI [1.009, 1.248], *P* = 0.034), renal dysfunction (OR = 1.263, 95% CI [1.086, 1.470], *P* = 0.002), elevated NT-proBNP (OR = 1.096, 95% CI [1.058, 1.136], *P* < 0.001), CTO (OR = 1.349, 95% CI [1.155, 1.574], *P* < 0.001), calcification (OR = 1.295, 95% CI [1.129, 1.485], *P* < 0.001), rotational atherectomy (OR = 3.261, 95% CI [2.351, 4.522], *P* < 0.001), LM PCI (OR = 1.384, 95% CI [1.111, 1.723], *P* = 0.004), multivessel PCI (OR = 1.318, 95% CI [1.117, 1.555], *P* = 0.001) and increased total stent length (OR = 1.017, 95% CI [1.015, 1.019], *P* < 0.001) were independent risk factors for the incidence of periprocedural myocardial infarction (shown in Model 3 in Table 2).

As described in Table 3, similar results were observed in logistic regression analysis for ADL with the incidence of periprocedural myocardial injury. After adjusting for age, renal dysfunction, NT-proBNP, CTO, calcification, rotational atherectomy, LM PCI, multivessel PCI and total stent length, multivariable logistic regression analysis demonstrated that impaired ADL was also an independent risk factor for the incidence of periprocedural myocardial injury (Model 3 in Table 3: OR = 1.131, 95% CI [1.017, 1.257], *P* = 0.023).

**Table 3:** Logistic regression analyses of ADL on periprocedural myocardial injury

	Variables	OR [95% CI]	*P* value
**Model 1**			
	impaired ADL	1.310 [1.188, 1.445]	< 0.001*
**Model 2**			
	impaired ADL	1.136 [1.026, 1.257]	0.014*
	Age	1.055 [0.978, 1.139]	0.167
	Renal dysfunction	1.409 [1.250, 1.588]	< 0.001*
	NT-proBNP	1.208 [1.158, 1.259]	< 0.001*
**Model 3**			
	impaired ADL	1.131 [1.017, 1.257]	0.023*
	Age (≥ 65)	1.069 [0.987, 1.158]	0.099
	Renal dysfunction	1.388 [1.225, 1.572]	< 0.001*
	NT-proBNP	1.166 [1.119, 1.216]	< 0.001*
	CTO	1.305 [1.143, 1.490]	< 0.001*
	Calcification	1.399 [1.251, 1.565]	< 0.001*
	Rotational atherectomy	3.347 [2.201, 5.091]	< 0.001*
	LM PCI	1.240 [1.022, 1.503]	0.029*
	Multivessel PCI	1.769 [1.533, 2.043]	< 0.001*
	Total stent length	1.017 [1.015, 1.019]	< 0.001*

Model 1 adjusted for none

Model 2 adjusted for age (years old), renal dysfunction (eGFR ≥ 60 or < 60 ml/min/1.73 m^2^), NT-proBNP (×10^3^pg/mL)

Model 3 based on Model 2, additionally adjusted for CTO (yes or no), calcification (yes or no), rotational atherectomy (yes or no), LM PCI (yes or no), multivessel PCI (yes or no) and total stent length (mm)

OR, Odds ratio; CI, Confidence interval. Other abbreviations as in Table 1. *P < 0.05

### The subgroup analysis

**Fig. 4:**
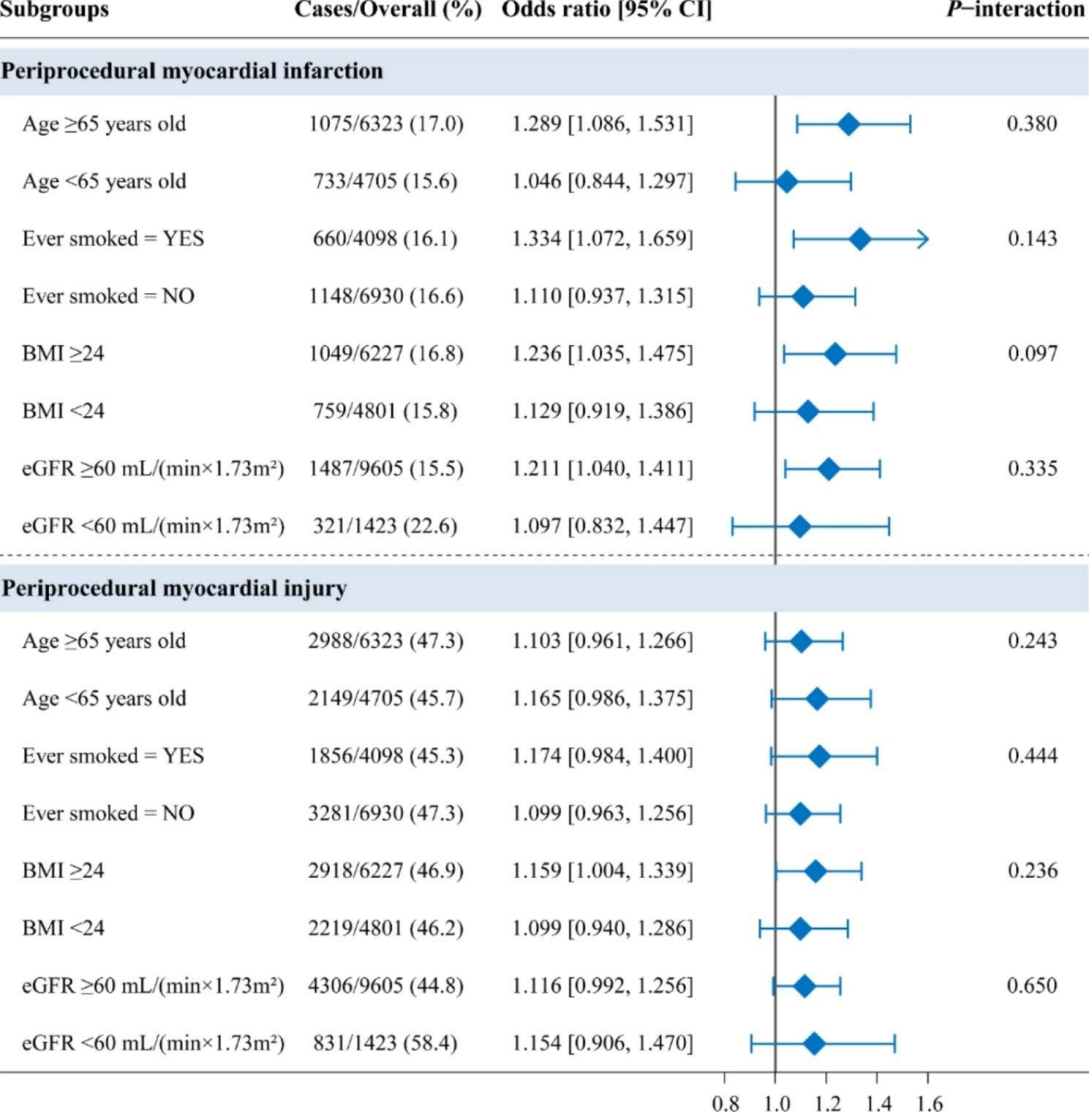
Forest plots of ADL for periprocedural myocardial injury and infarction in prespecified subgroups. In the subgroup analyses, multivariable logistic regression adjusted the same variables of Model 3 in Table 2. BMI, Body mass index; eGFR, estimated glomerular filtration rate

Figure 4 showed the exploratory analysis performed in subgroups based on age (≥ 65 or < 65 years old), ever smoked (yes or no), BMI (≥ 24 or < 24 kg/m^2^), and eGFR (≥ 60 or < 60 ml/min/1.73 m^2^). Impaired ADL was significantly associated with the increasing incidence of periprocedural myocardial infarction in age ≥ 65 years old (OR = 1.289, 95% CI [1.086, 1.531], *P* = 0.004), ever smoked (OR = 1.334, 95% CI [1.072, 1.659], *P* = 0.010), BMI ≥ 24 kg/m^2^ (OR = 1.236, 95% CI [1.035, 1.475], *P* = 0.019) and eGFR ≥ 60ml/min/1.73m^2^ (OR = 1.211, 95% CI [1.040, 1.411], *P* = 0.014) subgroups. The association between impaired ADL and periprocedural myocardial injury was significant only in BMI ≥ 24 kg/m^2^ (OR = 1.159, 95% CI [1.004, 1.339], *P* = 0.045) subgroup.

## Discussion

In this retrospective cross-sectional study, the morbidity of periprocedural myocardial infarction and injury was approximately 16.4% and 46.6%, respectively. The results revealed a negative correlation between BI scores and the incidence of periprocedural myocardial infarction and injury. Impaired ADL at hospital admission emerged as an independent risk factor for periprocedural myocardial injury and infarction. Exploratory analysis indicated that the association of impaired ADL with periprocedural myocardial infarction was more robust than with periprocedural myocardial injury. The current research emphasized the importance of assessing ADL scores at hospital admission for patients intending to undergo PCI. Physicians should be aware of the increased possibility of periprocedural myocardial infarction and injury events in patients with impaired ADL at hospital admission.

Periprocedural myocardial infarction and injury remain frequent life-threatening complications in cardiac revascularization, especially in PCI, as these complications are associated with the PCI procedure in cath lab settings [24]. Periprocedural myocardial infarction and injury in patients undergoing PCI are most commonly attributed to side branch occlusion [25, 26]. Moreover, their incidences are closely related to multiple factors, including aggressive stent expansion causing plaque rupture, stent distal embolization, no-reflow phenomenon, coronary dissection, coronary vasospasm, and failed procedures [27–29]. Therefore, early identification of periprocedural myocardial infarction and injury is crucial for deciding on appropriate therapeutic management. In addition to constant advancements in PCI technologies, preoperative assessment plays an important role in preventing and treating periprocedural myocardial infarction and injury.

ADL indicates individual self-care ability, encompassing activities such as self-feeding, toileting, and mobility. It does not function only as a tool for assessing the body’s condition, but also it serves as an indicator of overall health. There are several standard scales that are employed to estimate a patient’s ADL, including the Stanford Health Assessment Questionnaire Disability, the Katz index, and the BI [30–32]. The BI score has been found to be highly reliable, valid, and repeatable, rendering it particularly suitable for assessing a patient’s basic ADL upon hospital admission. Furthermore, the BI score can be utilized in emergency settings [33, 34]. ADL at hospital admission has primarily been used to predict the prognosis and survival duration of patients with neurological disorders and terminal cancers [31, 35]. It is widely acknowledged that higher ADL scores are strongly associated with better outcomes and fewer complications [35]. Impaired ADL has not only been associated with the incidence of cardiovascular diseases, such as heart failure and ACS, but also has been recognized as a prognostic factor for complications and long-term mortality in patients with these cardiovascular diseases [20, 30]. This study found impaired ADL was an independent risk factor for periprocedural myocardial infarction and injury. These findings are hoped to serve as a foundation for future research, fostering a deeper understanding of the impact of ADL on the risk of periprocedural myocardial infarction and injury, and offering insights for medical decision-making in the management of patients with periprocedural myocardial infarction and injury after PCI.

Impaired ADL often coexists with aging, inflammation, and organ dysfunction, including cardiac and renal dysfunction, which are well-established risk factors for periprocedural myocardial infarction [32]. A recent study revealed a correlation between cognitive decline and impaired physical function in elderly STEMI patients [36]. Moreover, the decline in cardiac and kidney function is known to contribute to the initiation and progression of periprocedural myocardial infarction and injury [6]. Specifically, the periprocedural myocardial infarction and injury patients in this study exhibited older age, higher WBC, higher BNP, and lower eGFR, consistent with findings from previous studies. Therefore, this study adjusted for multiple relevant confounding factors and conducted a multiple-factor logistic regression to prove that ADL serves as an independent risk factor, warranting adequate attention. It is crucial to develop effective methods of intervention to improve the ADL ability of periprocedural myocardial infarction and injury patients. Currently, relevant studies have demonstrated the potential of adopting aerobic exercise training to enhance ADL and improve the prognosis of patients with chronic heart failure [37]. This merits consideration for application in patients with periprocedural myocardial infarction and injury. Future studies and clinical efforts should prioritize successful improvement in ADL to decrease the incidence of periprocedural myocardial infarction and injury.

The relationship between ADL and periprocedural myocardial infarction and injury is multifactorial; however, the underlying mechanisms have yet to be fully elucidated [38]. A recent study has found that enhancing ADL can reduce the production of inflammatory cytokines, thereby attenuating the inflammatory response [39]. Periprocedural myocardial infarction and injury are linked to potential inflammatory responses associated with coronary artery disease and acute inflammatory reactions resulting from mechanical injury during PCI [40]. Therefore, enhancing ADL in patients may promote a shift towards a less pro-inflammatory state, potentially reducing the risk of periprocedural myocardial infarction and injury after PCI.

This study had several limitations that warrant further discussion. Firstly, like any retrospective research, it had inherent biases that could not be entirely avoided, such as confounding bias, selection bias, and information bias. Therefore, large-scale prospective studies need to be conducted to validate the findings. Secondly, the population distribution of the BI scores in this study was highly skewed, making it much more challenging to classify ADL into traditional categories of normal function (BI = 100), mild dysfunction (60–99), moderate dysfunction (40–59), severe dysfunction (20–39), and very severe dysfunction (BI < 20). This was attributed to the fact that the participants included in this study were all scheduled for elective PCI procedures, which compared to patients undergoing emergency PCI, generally had better self-care abilities. Consequently, the majority of patients exhibited normal ADL. Additionally, in the prevalent cultural practices of China and even Asia, individuals tend to conceal their symptoms of suffering [41], which may result in an overestimation of the prognosis for some patients with severely impaired ADL. Lastly, adjusting certain variables, such as stent diameter and the number of stents, was not feasible due to the study’s extensive time span, resulting in data loss.

## Conclusion

Impaired ADL at hospital admission was an independent risk factor for the incidence of periprocedural myocardial infarction and injury among patients following PCI. Along with the reduction in BI scores, there is a noted upward trend in the incidence of periprocedural myocardial infarction and injury. The results were significant for preventing and treating periprocedural myocardial infarction and injury in the clinic. Clinicians may consider integrating ADL assessment into the routine pre-procedural evaluation to effectively stratify patients and customize their management plans accordingly. Tailored monitoring protocols should be formulated for these high-risk patients before and after the procedure, including more frequent cardiac biomarker measurements and electrocardiographic monitoring, to ensure early detection and timely intervention.

### Electronic supplementary material

Below is the link to the electronic supplementary material.

**Additional File 1**: Baseline characteristics grouped by periprocedural myocardial injury or not.

## Data Availability

The datasets used and/or analyzed during the current study are available from the corresponding author on reasonable request.
