# Supplementary material for: Activities of daily living predict periprocedural myocardial infarction and injury following percutaneous coronary intervention: a cross-sectional study
Source: BMC Cardiovasc Disord. 2023 Aug 29;23:427. doi: 10.1186/s12872-023-03468-5 (PMC10466711; doi:10.1186/s12872-023-03468-5)
Supplement: Supplementary file 1 — Additional File 1: Baseline characteristics grouped by periprocedural myocardial injury or not. [file 12872_2023_3468_MOESM1_ESM.docx]

**Activities of Daily Living Predict Periprocedural Myocardial Infarction and Injury Following Percutaneous Coronary Intervention: A Cross-Sectional Study**

Yifan Pan^1^, Min Xu^1^, Yaosheng Mei^1^, Yunxiang Wang^1^, Qiongli Zhang^1,#^

^1^ Department of Cardiology, Yongkang People’s Hospital, Yongkang, Zhejiang 321300, China.

^#^ Qiongli Zhang is the corresponding author of this article.

**Correspondence**: Qiongli Zhang, MD, Ph.D.

Address: Department of Cardiology, Yongkang People’s Hospital, Yongkang, Zhejiang 321300, China.

E-mail: **[qlzhangyk@126.com](mailto:qlzhangyk@126.com)**

**Running title**: ADL predicts PMI

**Table S1 Baseline characteristics grouped by periprocedural myocardial injury or not.**

| **Characteristics** | Overall  (n=11028) | Periprocedural Myocardial Injury | | **P value** |
| --- | --- | --- | --- | --- |
|  |  | No (n=5891) | Yes (n=5137) |  |
| **Demographic features** |  |  |  |  |
| Age, years old | 65.8±10.4 | 65.6±10.3 | 66.0±10.5 | 0.034* |
| BMI, kg/m^2^ | 24.6±3.3 | 24.6±3.2 | 24.6±3.4 | 0.326 |
| Male, n (%) | 7854 (71.2) | 4238 (71.9) | 3616 (70.4) | 0.077 |
| Ever smoked, n (%) | 4098 (37.2) | 2242 (38.1) | 1856 (36.1) | 0.038* |
| Diabetes, n (%) | 3213 (29.1) | 1706 (29.0) | 1507 (29.3) | 0.679 |
| Hypertension, n (%) | 7496 (68.0) | 4032 (68.4) | 3464 (67.4) | 0.265 |
| Barthel index, scores | 97.0±8.7 | 97.4±8.0 | 96.5±9.4 | <0.001* |
| Impaired ADL, n (%) | 1943 (17.6) | 930 (15.8) | 1013 (19.7) | <0.001* |
| **PCI procedure data** |  |  |  |  |
| LM PCI, n (%) | 535 (4.9) | 213 (3.6) | 322 (6.3) | <0.001* |
| LAD PCI, n (%) | 5449 (49.4) | 2809 (47.7) | 2640 (51.4) | <0.001* |
| LCX PCI, n (%) | 2188 (19.8) | 1001 (17.0) | 1187 (23.1) | <0.001* |
| RCA PCI, n (%) | 2952 (26.8) | 1693 (28.7) | 1259 (24.5) | <0.001* |
| Multivessel PCI, n (%) | 1043 (9.5) | 364 (6.2) | 679 (13.2) | <0.001* |
| Rotational atherectomy, n (%) | 180 (1.6) | 29 (0.5) | 151 (2.9) | <0.001* |
| CTO, n (%) | 1162 (10.5) | 482 (8.2) | 680 (13.2) | <0.001* |
| Calcification, n (%) | 1775 (16.1) | 723 (12.3) | 1052 (20.5) | <0.001* |
| Total stent length, mm | 30.0 [18.0, 53.0] | 28.0 [16.0, 40.0] | 39.0 [23.0, 62.0] | <0.001* |
| **Laboratory data** |  |  |  |  |
| cTnI fold-elevation | 0.9 [0.1, 2.9] | 0.2 [0.0, 0.6] | 3.2 [1.7, 7.2] | <0.001* |
| WBC, ×10^9/L | 6.4±1.8 | 6.4±1.7 | 6.5±1.9 | <0.001* |
| LDL-C, mmol/L | 2.1±0.9 | 2.1±0.9 | 2.2±0.9 | 0.030* |
| CRP, mg/L | 1.3 [0.6, 3.2] | 1.2 [0.6, 2.8] | 1.5 [0.7, 3.6] | <0.001* |
| HbA1c, % | 6.1 [5.6, 6.8] | 6.10 [5.6, 6.9] | 6.0 [5.6, 6.8] | 0.019* |
| eGFR, mL/(min×1.73m^2^) | 87.0 [72.1, 97.7] | 89.0 [75.0, 99.0] | 85.0 [68.7, 95.9] | <0.001* |
| Platelet, ×10^9/L | 185.0 [152.0, 223.0] | 186.0 [153.0, 224.0] | 183.0 [150.0, 222.0] | 0.011* |
| NT-proBNP, pg/mL | 131.0 [57.0, 408.0] | 106.0 [49.0, 294.0] | 173.0 [70.0, 569.0] | <0.001* |
| **Mediccation** |  |  |  |  |
| ACEI/ARB , n (%) | 5583 (50.6) | 2928 (49.7) | 2655 (51.7) | 0.040* |
| BB, n (%) | 5493 (49.8) | 2871 (48.7) | 2622 (51.0) | 0.017* |
| CCB, n (%) | 4120 (37.4) | 2148 (36.5) | 1972 (38.4) | 0.039* |
| Statin, n (%) | 10849 (98.4) | 5785 (98.2) | 5064 (98.6) | 0.136 |

Categorical data are presented as n (%) and continuous data are expressed as mean ± standard deviation or median [interquartile range]. Other abbreviations as in Table 1. **P* < 0.05.
